# Swine Leukocyte Antigen-12 Behaves Like a Swine Leukocyte Antigen Classical Class I Protein and is a Potential Xenoantigen in Humans

**DOI:** 10.1097/TXD.0000000000001948

**Published:** 2026-04-21

**Authors:** Victor Novara Gennuso, Zheng Yu Wang, Luz M. Reyes, Matt Tector, A. Joseph Tector

**Affiliations:** 1 Department of Surgery, University of Miami School of Medicine, Miami, FL.; 2 Makana Therapeutics, Miami, FL.; 3 Founder Makana Therapeutics, Miami, FL.

## Abstract

**Background.:**

Using pigs as tissue donors may eliminate the shortage of replacement organs. As in allotransplantation, antidonor antibodies cause xenotransplant failure. Many antibodies target products of the major histocompatibility complex. Pigs contain a novel major histocompatibility complex gene, swine leukocyte antigen-12 (SLA-12), which has been proposed to behave as a classical class I SLA protein. Though predicted to bind B2m and short peptides, the functionality of the SLA-12 protein remains uncertain. In addition, its similarity to classical class I SLA molecules suggests that the SLA-12 could be a xenoantigen. Here we tested these predictions.

**Methods.:**

Protein modeling was used to compare SLA-12 and classical class I proteins SLA-1, SLA-2, and SLA-3. cDNA encoding an isoform of SLA-12 was introduced into a cell line devoid of HLA proteins. B2m binding of SLA-12 at the surface of this cell line was evaluated. In addition, expression of SLA-12 molecules was examined in these cells after disrupting the genes encoding the transporters of antigenic peptides for class I proteins. Serum samples collected from transplant patients were also tested for the presence of SLA-12-specific IgG and IgM.

**Results.:**

Predictive algorithms indicate SLA-12 and classical class I SLA proteins have very similar structures. We observed strong biochemical similarities as well in that SLA-12 binds to B2m and peptides to become a stable cell surface protein. Finally, we observed that SLA-12-expressing cells bind more serum IgG and IgM from some transplant patients, indicating that it can be a xenoantigen.

**Conclusions.:**

SLA-12 has marked structural and biochemical similarities with classical class I SLA proteins and may be another target of human anti-pig antibodies. Similar approaches may be successful in mitigating the antigenic issues of SLA-12, SLA-1, SLA-2, and SLA-3.

## INTRODUCTION

Classical leukocyte antigen proteins are encoded by genes in the major histocompatibility complex (MHC) and play a key role in cellular immunity. These molecules bind and display endogenous and exogenous peptides that the T-lymphocytes interact with via the T-cell receptor. Often, encountering foreign peptide, such as those originating from pathogens or cancer, leads to the T-cell receptor delivering activating signals to the T cell. When functioning normally, these processes provide protection against various infectious diseases and neoplasms.

Leukocyte antigens can be problematic in the setting of organ transplantation because they are extremely polymorphic throughout the population. Consequently, most donor organs express allelic variants of MHC proteins that are foreign to the tissue recipient. These exogenous leukocyte antigens can activate recipient T cells because they mimic endogenous MHC proteins complexed with pathogen-derived peptides. This challenge is managed by pairing donors and recipients to minimize their MHC disparities and by inhibiting T-cell activation through various pharmaceutical approaches.^1^

Finding ideal donor-recipient matches is difficult in human transplantation because in each haploid genome there are multiple loci that encode the HLAs. Three distinct genes encode 3 gene class I HLA (HLA-A, -B, and -C) and 6 genes synthesize class II HLA (-DRa/b, -DQa/b, and -DPa/b). Some HLA genes have only a few allelic variants, whereas others have >100. The polymorphic nature of HLA further complicates allotransplantation because a foreign HLA allele can not only activate T cells but also serve as targets for B-lymphocyte activation.^2,3^ Once stimulated, B cells can produce high levels of HLA-specific antibodies that can damage foreign tissues. These mechanisms include activation of the complement cascade and directing innate immune-cell cytotoxicity toward antibody-coated cells.^4-6^

Although issues of HLA disparity must be accounted for in organ transplantation, they are not the major challenge to this therapy. The greatest challenge arises from the lack of human donor organs, which prevents most patients from having access to this treatment. Consequently, animals, in particular pigs, are being studied as a potential source of abundant organs for humans. Xenotransplantation experiences similar immunologic hurdles to allotransplantation in that recipient antidonor cellular and humoral immunity attacks the transplanted pig tissue. Pig-to-nonhuman primate xenotransplant models demonstrate that inhibiting the CD40/CD154 costimulatory pathway is critical for long-term organ function.^7-9^ The need for costimulatory blockade is likely 2-fold. First, primate CD4 and CD8 cells activate and perform cell-mediated lympholysis when exposed to pig cells.^10-15^ Second, the formation of de novo anti-pig antibodies must be suppressed to avoid antibody-mediated rejection.^16^ The first attempts at human xenotransplantation have also described rejection consistent with cellular and/or humoral immune activity.^17-23^

Humoral immunity initially caused rapid graft destruction because all humans have antibodies to glycans found on pig tissues. However, genetic engineering has enabled the development of multigene knockout pigs that no longer express several carbohydrate xenoantigens.^24-28^ Reducing pig antigenicity and inhibiting costimulatory pathways have extended survival in the kidney xenotransplant model to >1 y.^29,30^ The fact that gene-edited pigs exhibit even lower antigenicity toward humans than they do toward nonhuman primates increases optimism for clinical success.^31^ The best donor pig for humans is a 3 gene knockout because 30% of transplant patients have few antibodies toward 3 gene knockout cells.^32^ These knockouts eliminate multiple pig-specific carbohydrates which trigger human anti-pig antibodies.

The swine leukocyte antigens (SLAs), homologs of HLA, can also be targeted by human antibodies. Both classical class I SLA (-1, -2, -3) and class II SLA (-DR, -DQ) can be targets of human IgG and IgM^.32,33–36^ A fourth potential class I SLA gene, SLA-12, has been proposed on the basis of genomic DNA sequencing.^37,38^ This locus produces mRNA transcripts; however, the molecule may be nonfunctional because of its short cytoplasmic tail.^37,38^ Like classical class I SLA (SLA-1, -2, -3), we demonstrate that SLA-12 binds B2-microglobulin (B2m) and peptides for display at the cell surface. SLA-12 also mimics other class I SLA because it can be a xenoantigen targeted by human antibodies.

## MATERIALS AND METHODS

### SLA-12 Structural Analysis

The predicted structure of SLA-12*01:01 was developed using the AlphaFold program by Google DeepMind.^25^ The signal sequence was removed before running the structural prediction. PyMol software (The PyMOL Molecular Graphics System, Version 3.0 Schrödinger, LLC.) was then used to analyze and align the SLA-12 protein to other class I SLA protein structures that have associated crystal structures for comparison. The following crystal structures used for SLA-1, PDB#6LF9; SLA-2, PDB#8GQW; and SLA-3, PDB#5H94.^39–41^ Root-mean-square distances for each alignment were calculated in PyMOL and used as an evaluation of structural similarities.

### Flow Cytometry

The following antibodies were used: anti-pig SLA class I (clone JM1E3, Invitrogen); anti–B2m antibody (clone 2M2; BioLegend, San Diego, CA). These antibodies were directly labeled with fluorescein isothiocyanate. Phenotypic analysis of sorted cells was performed using either an Accuri C6 or a FACSLyric flow cytometer (Becton Dickinson). Flow sorting to isolate transporter of antigenic peptides (TAP)-deficient expressing cells was performed using a 4-laser BD FACSAria Fusion running FACSDiva 8 (version 8.0.2) located in the Sylvester Comprehensive Cancer Center Flow Cytometry Shared Resource at the University of Miami Miller School of Medicine. The population of interest was initially gated using Forward (FSC) and side scatter (SSC), and doublets were excluded using both FSC-A × FSC-W and SSC-H and SSC-W dot blots. This gating strategy was used in all flow cytometry experiments described in this report.

### Cell Lines

The base cell line of this study is C1R, a human B lymphoblastoid cell line that was previously genetically engineered to eliminate multiple surface molecules (class I HLA and class II HLA, Fc receptor, and IgG).^42^ Gene-edited C1R was cultured in Roswell Park Memorial Institute 1640 Medium (1X) (Cytiva) supplemented with 10% heat-inactivated fetal bovine serum (Cytiva), 1% penicillin-streptomycin (Cytiva), and Normocin (50 mg/mL) (InvivoGen).

To drive expression of SLA-12, the open-reading frame corresponding to allele SLA-12*01:01 in the Immuno Polymorphism Database for MHC genes (accession No. SLA09721; http://www.ebi.ac.uk/ipd/mhc/)^43,44^ was cloned into the PREP4 expression vector (Fisher Scientific). Gene-edited C1R cells demonstrating increased cell-surface B2m expression after transfection were sorted by flow cytometry as noted previously and maintained with hygromycin at 400 μg/mL (Invitrogen).

C1R cells expressing SLA-12 were electroporated in media containing recombinant Cas12a (Integrated DNA Technologies) assembled with guide RNA (gRNA) specific for TAP1 (NCBI Reference Sequence: NM_000593.6; 5′-AAGCCATTAGCTGCGGCACTG-3′) and TAP2 (NCBI GenBank: OR643831.1; 5′-TGGGGACACTGCTGCTCCCGC-3′) genes. Cells demonstrating reduced SLA-12 expression were flow sorted using the strategy described previously.

To validate the TAP knockouts, DNA sequencing analysis of gRNA/Cas12-targeted TAP1/TAP2 genomic regions was performed as follows: genomic DNA from the engineered and wild-type cells was extracted using the QIAGEN DNeasy Blood and Tissue Kit. Polymerase chain reaction (PCR) was performed to amplify regions flanking the editing targets in TAP1/2. The primers (TAP1 forward: 5′- AGTACTGCTACTTCTCGCCGACTGGGTGCT-3′; TAP1 reverse: 5′-TGCGGGCAGTGCCGCTGCATAACTGACAACGAA-3′; TAP2 forward: 5′- CGTATCCGTTGACAGAGCCA-3′; TAP2 reverse: 5′-GGCCCTTTTACCACCTCCAA- 3′) were designed to flank the gRNA/Cas12 target sites and amplify 376 bp for TAP1 and 557 bp for TAP2. Human TAP1/2 PCR products were purified with the QIAGEN MinElute PCR Purification Kit and ligated into pCR2.1-TOPO plasmid using the TOPO TA cloning kit (Invitrogen, Carlsbad, CA) and analyzed by Sanger sequencing (Genewiz Inc, South Plainfield, NJ) and the NCBI BLAST algorithm (22). TAP sequencing results are presented as **Figure S1** (**SDC,** https://links.lww.com/TXD/A858).

### Temperature Shift Experiment

Gene-edited C1R cells were cultured continuously at 37 °C in a humidified 5% CO_2_ incubator. For low-temperature culture, cells were cultured in a polypropylene 50 mL conical tube (Falcon) and placed in a water bath at 26 °C for 24 h. Flow cytometric analysis of B2m and class I SLA antibody binding was performed using the antibodies and flow cytometry protocol described previously. Median fluorescence intensity for the vector-only cells was subtracted from the SLA-12-expressing but TAP-deficient cells.

### Crossmatching With Human Sera

Collection of the sera samples from patients providing written informed consent was approved by the institutional review board (IRB No.: 20191199). Samples were collected from adult transplant patients undergoing care in the solid organ transplant program. The collection was not limited to any specific organ. The HLA sensitization status of each patient was noted to facilitate sorting samples into those containing ant-HLA antibodies and those lacking them. Cells can acquire xenoantigens from cell culture media components, thus raising background signal when crossmatching. To minimize this, the cells were grown in the absence of serum. Next, cells were resuspended in phosphate buffered saline and incubated with human serum at a final concentration of 25% by volume. After washing away unbound proteins, the cells were incubated with either fluorescein isothiocyanate goat antihuman IgG (catalog no. 109-096-098, Jackson ImmunoResearch Laboratories Inc.) or Alexa Fluor 488 goat antihuman IgM (catalog no. 109-546-129, Jackson ImmunoResearch Laboratories Inc.). Cells were washed again, fixed in 4% paraformaldehyde, and analyzed using a FACSLyric flow cytomter (Becton Dickinson) and the FlowJo analysis program.

## RESULTS

**FIGURE 1:**
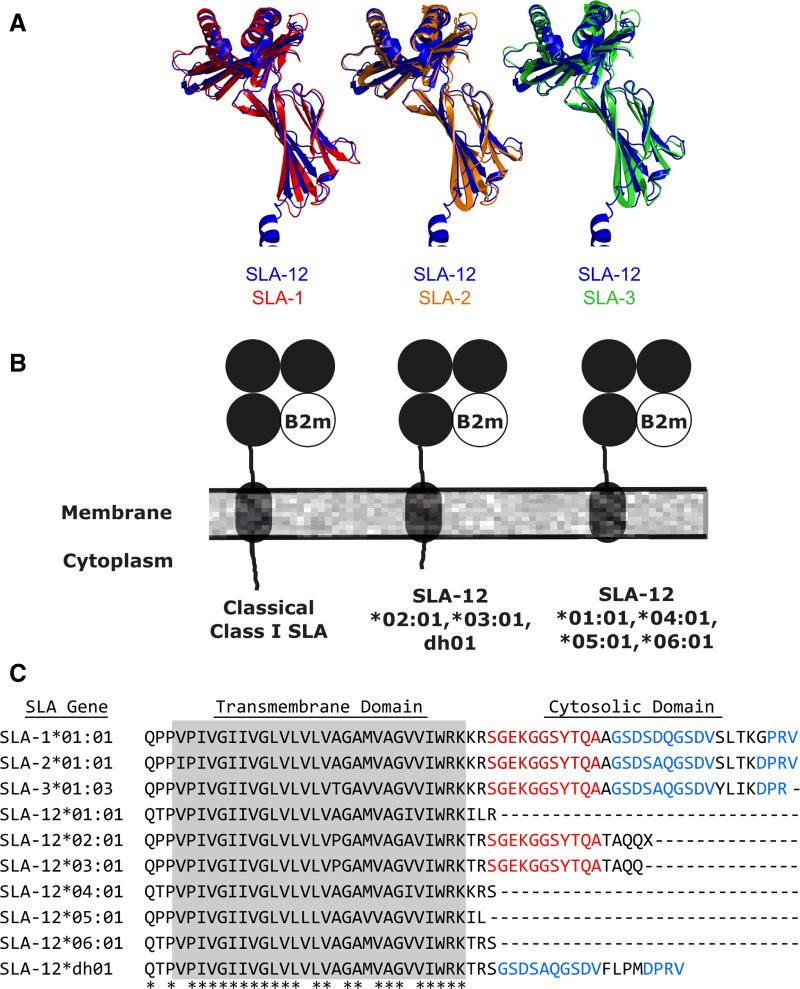
. Comparing SLA-12 and classical class I SLA proteins. A, An alpha fold prediction of the extracellular domain of SLA-12 (blue structure) was overlayed with classical class I SLA crystal structures (SLA-1, red; SLA-2, gold; SLA-3, green). B, Schematic highlighting the differences in the C-termini of classical class I SLA and different SLA-12 alleles. C, Transmembrane and cytoplasmic sequences of the seven available SLA-12 alleles (SLA-12*01:01, -12*02:01, -12*03:01, -12*04:01, -12*05:01, -12*16:01, and -12*dh01) were aligned against the corresponding sequences from classical molecules SLA-1*01:01, SLA-2*01:01, and SLA-3*03:01. The gray box highlights the transmembrane domains with asterisks representing residues where all analyzed chains contain the same amino acid. Red and blue letters highlight regions of high similarity between classical class I SLA proteins and the SLA-12 alleles. SLA, swine leukocyte antigen.

SLA-12 is a class I–like gene found in several pig genomes. Although SLA-12 transcription has been detected, early reports found its open-reading frame encodes a protein with an uncharacteristically short cytoplasmic tail relative to classical class I SLA proteins.^37^ We examined the predicted amino acid sequences of the 6 SLA-12 alleles (SLA-12*01:01, 02:01, 03:01, 04:01, 05:01, 06:01) found in the Immuno Polymorphism Database for MHC genes database and another allele reported in the National Center for Biotechnology Database (SLA-12*dh01, NCBI accession #: FJ883494.1). Sequence comparisons of these molecules with classical class I SLA proteins reveal approximately 87% identity in their extracellular domains (data not shown). We compared the predicted SLA-12 structure, generated by alpha fold, with SLA-1, SLA-2, and SLA-3 proteins that have solved crystal structures (Figure 1A). This demonstrated high structural homology with the root mean square distances being 0.971Å (SLA-12 versus SLA-1), 1.547 Å (SLA-12 versus SLA-2), and 1.216 Å (SLA-12 versus SLA-3). These data suggest that SLA-12 is likely associated with the B2m subunit, as well as peptide fragments, much like classical class I SLA.

Further analysis of 7 identified SLA-12 alleles indicated that the cytoplasmic tails showed the greatest difference between SLA-12 and classical class I SLA proteins (Figure 1B and C). The transmembrane and cytoplasmic domains of those proteins are shown in Figure 1B. The same domains from classical class I SLA molecules SLA-1*01:01, SLA-2*01:01, and SLA-3*03:01 are shown for comparison. All SLA-12 alleles have truncated cytoplasmic domains, with some being shorter than others. Notably, the residual cytoplasmic tails of SLA-12*02:01, SLA-12*03:01, and SLA-12*dh01 have partial sequence homology with classical class I proteins. In contrast, SLA-12*01:01, SLA-12*04:01, and SLA-12*05:01 all encode proteins containing only a few cytosolic amino acids. The subsequent experiments examined SLA-12*01:01, a molecule lacking a cytosolic tail, as that was representative of the greatest number of SLA-12 alleles and the largest difference from classical proteins.

**FIGURE 2:**
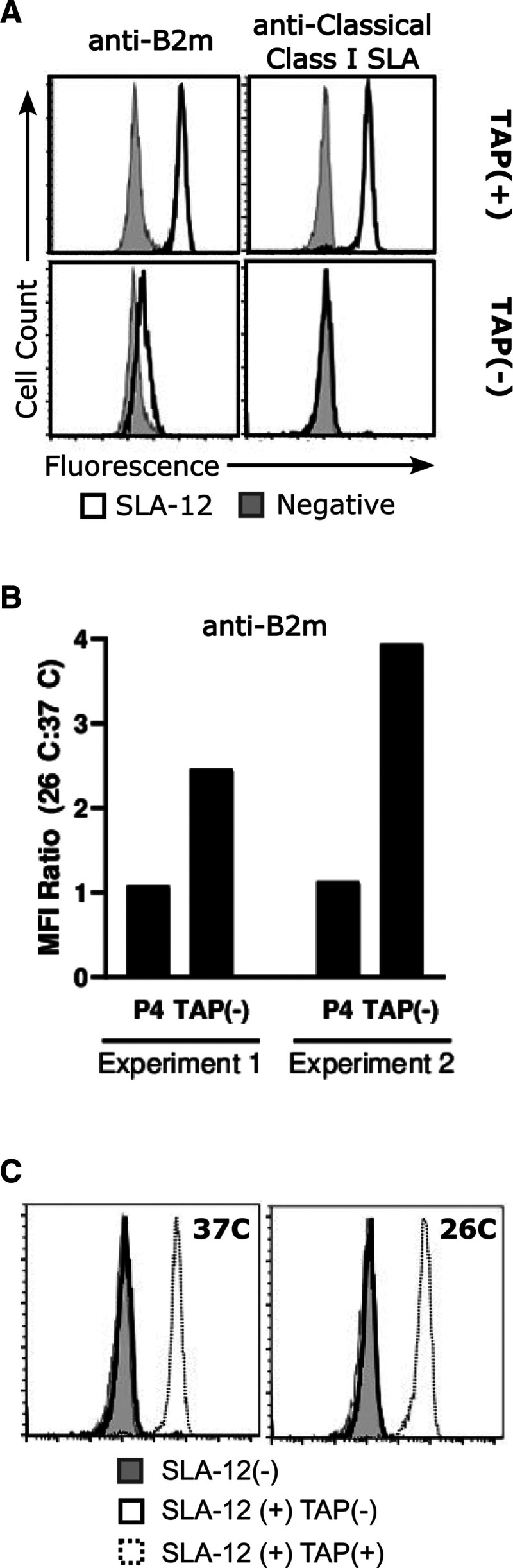
. A peptide source is required for cell surface expression of B2m-associated SLA-12. A, Human cells transfected with the SLA-12*01:01 open-reading frame were probed with monoclonal antibodies toward classical class 1 SLA proteins (upper right panel) or the B2m subunit (upper left panel). The same cell containing the expression vector minus the SLA-12 open-reading frame was similarly analyzed as a negative control for nonspecific antibody binding. Gray histograms represent negative control and white histograms represent the binding of the B2m and the class I SLA antibodies. The lower panels represent a repeat of this experiment after the cells had been edited to inactivate TAP genes. B, The TAP-deficient cells expressing SLA-12 were incubated at 26 °C and 37 °C overnight. The cells were stained with anti-B2m antibodies, with MFI determined by flow cytometry. The ratio for staining was calculated by dividing the MFI value at 37 °C by the MFI value at 26 °C. Two experiments are shown. Cells containing an empty expression vector (P4) are shown as negative controls. C, The experiment in (B) was repeated except that cells were stained with an antibody against classical class I SLA proteins. Gray histograms represent the empty vector negative control SLA-12(–); The white histogram with black outline represents the SLA-12 expressing but TAP-deficient cells SLA-12(+)TAP(–); The dotted line histogram represents the staining for SLA-12 expression in cells having functional TAP genes SLA-12(+) TAP(+). MFI, median fluorescence intensity; SLA, swine leukocyte antigen; TAP = transporter of antigenic peptide.

The open-reading frame of SLA-12*01:01 was cloned into a mammalian expression vector and introduced into a human cell line engineered to be devoid of class I and class II HLA.^42^ We then examined whether this cell line expressed an SLA-12 protein at the cell surface by performing flow cytometry. Two antibodies were used, 1 specific to classical class I SLA proteins and the second which targets the B2m subunit. Cells containing the SLA-12 expression vector bound markedly more of each antibody than the same cell line carrying a vector-only control (Figure 2A). Neither of these antibodies bound the cells in the absence of SLA-12, verifying the presence of an SLA-12/B2m complex.

In addition to B2m, classical class I SLA proteins bind short peptides.^39–41^ These peptides are supplied to class I proteins by TAP complexes.^42^ The TAP transporter consists of 2 subunits, TAP1 and TAP2, which move peptides from the cytosol to the secretory pathway.^45^ After peptides associate with class I and B2m proteins, the trimolecular complex is released for transit to the cell surface. Consequently, cells lacking TAP activity also fail to express high levels of class I proteins.^46,47^

We examined cells with and without functional TAP to determine whether SLA-12 required a peptide supply for cell-surface expression. SLA-12 positive cells were treated with CRISPR endonucleases targeting TAP1 and TAP2 genes. The class I and B2m antibodies enabled sorting of SLA-12-negative cells from SLA-12-positive cells by flow cytometry. The TAP1 and TAP2 genes from these bulk-sorted and SLA-12-deficient cells were sequenced (**Figure S1, SDC**, https://links.lww.com/TXD/A858). TAP1 gene sequences revealed a mixture of unedited TAP1 genes as well as the presence of various insertions and deletions. The TAP2 analysis showed a mixture of gene deletions with no unedited sequences. The variety of gene sequences was expected, given that bulk sorting yields polyclonal cell populations. Nevertheless, many cells appear to contain disrupted TAP1, and the entire cell population lacks functional TAP2. Despite this compelling result, a loss of SLA-12 expression during the editing process could also yield this data. To eliminate this possibility, we incubated the edited cells at low temperature to see whether SLA-12 expression returned. We reasoned that, if present, SLA-12 stability may be enhanced at low-temperature incubation, allowing it to reach the surface in TAP-deficient cells. This phenomenon has been observed in TAP-deficient mouse cells, where class I protein expression is lost at 37 °C but returns at 26 °C.^48^ Reactivity of the B2m antibody increased in TAP-deficient cells carrying the SLA-12 expression vector after reduced temperature incubation (Figure 2B). Cells containing the empty expression vector did not reproduce this phenomenon, demonstrating that the SLA-12 protein was essential for signal. Unlike B2m, the class I SLA antibody binding did not increase at low temperature (Figure 2C). Presumably, without a peptide source, the SLA-12 molecules do not acquire the epitope recognized by the class I antibody.

**FIGURE 3:**
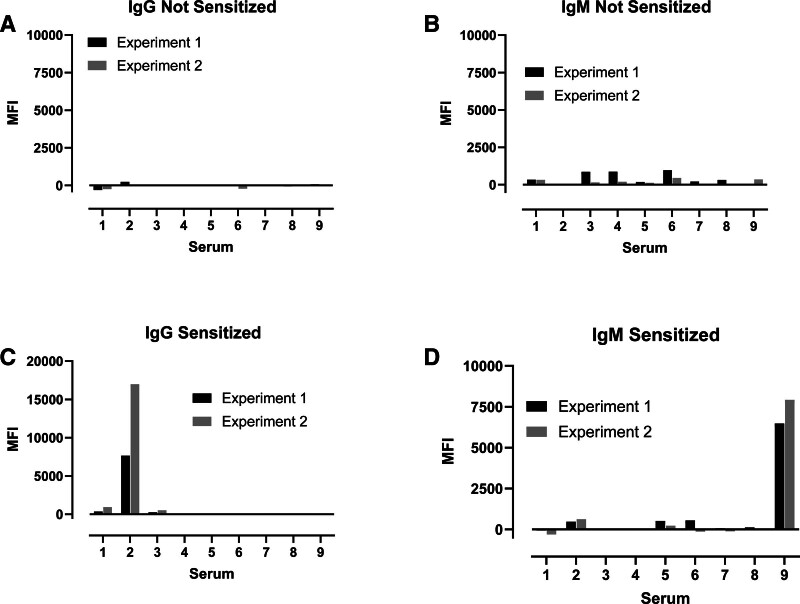
. Human IgG and IgM can bind SLA-12-expressing cells. SLA-12 expressing TAP-positive cells were incubated with human sera followed by fluorescent antibodies that detect either human IgG or IgM. Nine sera were from patients lacking antibodies to class I HLA proteins (IgG (A) and IgM (B)). The other 9 sera were from patients known to have IgG antibodies that recognize class I HLA proteins (IgG (C) and IgM (D)). SLA-12-deficient cells were subjected to the same analysis. MFI values were calculated by flow cytometry. Plots show values obtained by subtracting the negative control MFI from the MFI of cells expressing cell surface SLA-12. The experiment was repeated twice. MFI, median fluorescence intensity; SLA, swine leukocyte antigen; TAP = transporter of antigenic peptide.

We next examined whether human antibodies also recognized SLA-12, given its similarity to the xenoantigenic classical class I SLA proteins. First, we split human serum samples into 2 categories: (1) those lacking HLA-specific antibodies (Figure 3A and B), and (2) those having antibodies to class I HLA proteins (Figure 3C and D). Sera were categorized in this manner because anti-HLA antibodies can increase the frequency of SLA reactivity. Zero of 9 sera lacking HLA antibodies had IgG or IgM that recognized cells expressing SLA-12. In contrast, one of the 9 sera containing HLA antibodies exhibited IgG binding to SLA-12-positive cells, and a different serum contained SLA-12-specific IgM.

## DISCUSSION

As xenotransplantation is moving toward clinical application, it will be critical to be able to evaluate candidate xenoantigens in detail. The identification of HLA antigens and the development of pretransplant screening technologies have been incredibly important to improving results in clinical allotransplantation. Xenotransplantation will similarly benefit from clarifications on which SLA antigens are expressed in pig cells, and from the development of sensitive assays to detect specific anti-pig and more specifically anti-SLA antibodies. Our results demonstrate that the open-reading frame of SLA-12 encodes a cell-surface membrane protein that contributes to humoral xenoantigenicity. This molecule requires a peptide supply to reach the cell surface. As expected from its sequence homology to other class I proteins, B2m is part of the mature trimolecular complex.

Though we do not examine the ability of SLA-12 to stimulate T cells in this report, we expect it to participate in cellular immunity. Most notably, it has high sequence identity with other class I SLA proteins and requires the peptide to be expressed. Consequently, we anticipate future studies will find that SLA-12 stimulates human anti-pig cellular immunity. In addition, we expect this protein to also contribute to protective immunity in the pig by displaying pathogen-derived peptides to CD8 cells. Whether SLA-12 participates in natural killer cell recognition like other classical and nonclassical class I proteins do, will also require further investigation.

The most obvious difference between the SLA-12 molecules and classical class I SLA proteins occurs at their C terminus. Three alleles (SLA-12*02:01, SLA-12*03:01, and SLA-12*dh01; Figure 1) have vestiges of the tails found on classical class I proteins. We hypothesize that these cytosolic tails will not hinder the ability of those molecules to assemble with B2m and peptide in the lumen of the endoplasmic reticulum. Furthermore, these C-terminal sequences do not contain obvious motifs known to prevent trafficking of molecules through the secretory pathway. Therefore, SLA-12*02:01, SLA-12*03:01, and SLA-12*dh01 should be able to reach the cell surface. The other 4 alleles (SLA-12*01:01, SLA-12*04:01, SLA-12*05:01, and SLA-12*0 6:01) lack a cytoplasmic tail. Nevertheless, the SLA-12*01:01 molecule assembled with B2m and peptides and was recognized by a conformation-dependent antibody at the cell surface. The intracellular portions of class I homologs in other species have several roles: (1) they drive internalization of the protein from the cell surface^49^; (2) they alter the diffusion of the protein in the plasma membrane^50^; and (3) they can be targeted by viral machinery to alter cell surface expression and bypass protective immunity.^51,52^ Future work will be needed to determine whether the lack of a cytoplasmic tail affects the mobility of SLA-12 in the cell. It will also be interesting to learn whether the loss of C-terminal sequences helps SLA-12 overcome viral defense mechanisms.

Future studies will help to understand the extent to which SLA-12 contributes to cellular xenoantigenicity. First, does the gene’s activity change under different conditions, such as inflammatory situations? Second, does protein expression fluctuate for reasons unrelated to the abundance of SLA-12 transcripts? Third, does SLA-12 interact with receptors on T cells and natural killer cells to regulate their activation?

Although we demonstrate its potential role as a humoral xenoantigen, the SLA-12 gene does not appear to be as highly transcribed as other class I SLA.^53^ Because peptides and B2m bind other class I proteins, SLA-12 must compete for these resources, which may affect its cell-surface abundance. Finally, the relative importance of SLA-12 as a xenoantigen will rely in part on which cells and tissues express this protein. The reductionist approach we used for this first study cannot address these issues. Creating SLA-12-specific detection reagents, such as monoclonal antibodies, will enable further study in pig cells and tissues.

## Supplementary Material


