# Supplementary material for: Swine Leukocyte Antigen-12 Behaves Like a Swine Leukocyte Antigen Classical Class I Protein and is a Potential Xenoantigen in Humans
Source: Transplant Direct. 2026 Apr 21;12(5):e1948. doi: 10.1097/TXD.0000000000001948 (PMC13102430; doi:10.1097/TXD.0000000000001948)
Supplement: Supplementary file 1 [file txd-12-e1948-s001.pdf]

A	G	C	C	A	T	T	A	G	C	T	G	C	G	G	C	A	C	T	G	G	Number of Clones	Mutation
•	•	•	•	•	•	•	•	•	•	•	•	•	•	•	•	•	•	•	•	•	9	none
•	•	•	•	•	•	•	•	•	•	•	•	G	•	•	•	•	•	•	•	•	1	point
•	•	•	•	•	•	•	•	•	•	•	•	•	•	•	•	•	N	G	•	•	1	point
•	•	•	•	•	•	•	•	•	•	•	•	•	•	•	•	•	-	•	•	•	1	deletion
•	•	•	•	•	•	•	•	•	•	•	•	•	•	•	•	•	•	•	-	•	2	deletion
•	•	•	•	•	•	•	•	•	•	•	•	•	•	•	•	•	-	-	-	-	2	deletion
•	•	•	•	•	•	•	•	•	•	•	•	•	-	-	-	-	-	-	•	•	1	deletion
•	•	•	•	•	•	•	•	•	•	•	•	•	-	-	-	-	-	-	-	•	2	deletion
•	•	•	-	-	•	-	•	-	-	•	-	-	-	-	-	•	-	-	-	-	1	deletion

A	T	T	A	G	C	T	G	C	G	G	C	A		C				T	G	G	Number of Clones	Mutation
•	•	•	•	•	•	•	•	•	•	•	•	•	•	•	T	G	G	•	•	•	1	insertion
•	•	•	•	•	•	•	•	•	•	•	•	•	C	•	•	•	•	•	•	•	1	insertion

TAP1 Sequencing

T	G	T	G	G	G	G	A	C	A	C	T	G	C	T	G	C	T	C	C	C	G	C	T	C	T	G	T	C	T	G	G	C	C	A	C	C	Number of Clones	Mutation
•	•	•	•	•	•	•	•	•	•	•	•	•	•	•	-	-	-	-	-	-	•	•	•	•	•	•	•	•	•	•	•	•	•	•	•	•	1	deletion
•	•	•	•	•	•	•	•	•	•	•	•	•	•	•	-	-	-	-	-	•	-	•	-	•	•	•	•	•	•	•	•	•	•	•	•	•	1	deletion
•	•	•	•	•	•	•	•	•	•	•	•	•	•	•	-	-	-	-	-	-	-	-	•	•	•	•	•	•	•	•	•	•	•	•	•	•	1	deletion
•	•	•	•	•	-	-	-	-	-	-	-	-	-	-	-	-	-	-	-	-	-	-	-	•	•	•	•	•	•	•	•	•	•	•	•	•	1	deletion
•	•	•	•	T	•	•	C	•	•	•	-	-	•	-	-	•	-	•	•	-	-	-	-	-	-	-	-	-	-	-	-	-	-	-	-	-	1	deletion
•	•	-	-	-	-	-	-	-	-	-	-	-	-	-	-	-	-	-	-	-	-	-	-	-	-	-	-	-	-	-	-	•	•	•	•	•	1	deletion
•	•	•	•	•	•	•	•	•	•	•	-	-	-	-	-	-	-	-	-	-	-	-	-	-	-	-	-	-	-	-	-	-	-	-	-	•	1	deletion

TAP 2 Sequencing
